# Structural basis of nanobody-mediated blocking of BtuF, the cognate substrate-binding protein of the *Escherichia coli* vitamin B12 transporter BtuCD

**DOI:** 10.1038/s41598-017-14512-8

**Published:** 2017-10-30

**Authors:** S. A. Mireku, M. M. Sauer, R. Glockshuber, K. P. Locher

**Affiliations:** Institute of Molecular Biology and Biophysics, Eidgenössische Technische Hochschule (ETH) Zürich, CH-8093 Zürich, Switzerland

## Abstract

Bacterial ABC importers catalyze the uptake of essential nutrients including transition metals and metal-containing co-factors. Recently, an IgG antibody targeting the external binding protein of the *Staphylococcus aureus* Mn(II) ABC importer was reported to inhibit transport activity and reduce bacterial cell growth. We here explored the possibility of using alpaca-derived nanobodies to inhibit the vitamin B12 transporter of *Escherichia coli*, BtuCD-F, as a model system by generating nanobodies against the periplasmic binding protein BtuF. We isolated six nanobodies that competed with B12 for binding to BtuF, with inhibition constants between 10^−6^ and 10^−9^ M. Kinetic characterization of the nanobody-BtuF interactions revealed dissociation half-lives between 1.6 and 6 minutes and fast association rates between 10^4^ and 10^6^ M^−1^s^−1^. For the tightest-binding nanobody, we observed a reduction of *in vitro* transport activity of BtuCD-F when an excess of nanobody over B12 was used. The structure of BtuF in complex with the most effective nanobody Nb9 revealed the molecular basis of its inhibitory function. The CDR3 loop of Nb9 reached into the substrate-binding pocket of BtuF, preventing both B12 binding and BtuCD-F complex formation. Our results suggest that nanobodies can mediate ABC importer inhibition, providing an opportunity for novel antibiotic strategies.

## Introduction

ATP-binding cassette (ABC) importers are multi-subunit membrane protein complexes mediating the uptake of essential nutrients^1^. They contain a soluble or membrane-anchored substrate binding protein (SBP) that recognizes the substrate and delivers it to the transporter located in the cytoplasmic membrane^2^. Recently, an approach to inhibit transport by an ABC importer was established by blocking the interaction of the SBP with the transporter using a Fab fragment of an IgG antibody that specifically bound to the SBP and thus restricted the interaction with the transporter by steric hindrance. This study was performed with the *Staphylococcus aureus* SBP MntC, which is part of the transporter system responsible for the uptake of the essential nutrient Mn(II)^3^. We hypothesized that nanobodies, single chain variable domain antibody fragments derived from heavy chain only antibodies of camelids, might be able to accomplish similar blocking^4^. This would offer additional possibilities in developing novel antibiotic strategies, because nanobodies are less immunogenic and smaller than antibodies, thus offering certain advantages for therapeutic approaches.

The ABC importer BtuCD-F catalyzes vitamin B12 (cyanocobalamin or Cbl) and cobinamide uptake into the cytoplasm of *Escherichia coli* ^5,6^. Like other members of the ABC transporter superfamily, BtuCD-F couples the energy of ATP binding and hydrolysis to substrate translocation across lipid bilayers^7^. ABC transporters share a common architecture of two transmembrane domains and two nucleotide binding domains (NBDs)^8,9^, but large scale mechanistic diversity has been observed^10^. In BtuCD-F, the BtuC subunits are membrane-bound, the BtuD subunits are the NBDs, while BtuF is the periplasmic SBP^11–14^. Crystal structures of BtuCD-F representing distinct states of the transport cycle and extensive functional studies have provided a detailed description of the transport mechanism^15–18^. Because BtuCD-F is a well characterized transporter, we found it suitable as a model to study functional inhibition.

To explore the functional inhibition by blocking the SBP with a nanobody, we generated BtuF-specific nanobodies that act as competitive inhibitors of B12 binding to BtuF and investigated their effects on BtuCD-F mediated B12 import *in vitro*. We singled out a particularly promising, inhibitory nanobody and determined its structure in complex with BtuF, revealing the molecular basis of inhibition. Our data showed that a nanobody, which is much smaller than a Fab fragment, could also mediate inhibition of B12 import by competing with substrate binding and transport.

## Results

### Selection of BtuF-specific nanobodies

**Figure 1:**
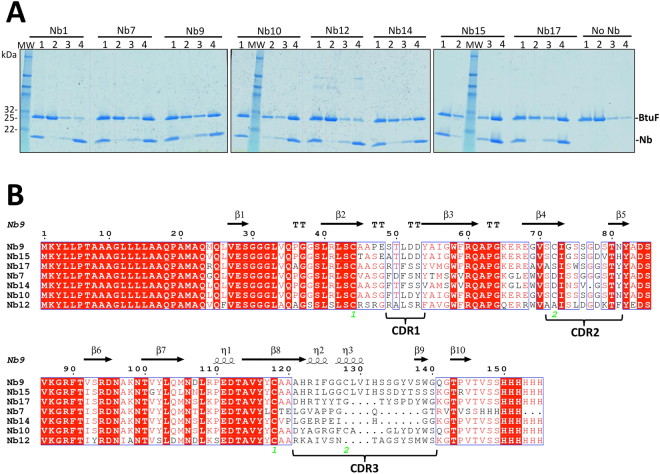
Selection of BtuF-specific nanobodies and sequence alignment. (**A**) SDS-PAGE analysis of pull-down assays. His-tagged nanobodies (Nb) were immobilized on Ni-NTA beads and mixed with a 1.5 - fold molar excess of tag-less BtuF. Controls were performed by omission of nanobody (no Nb) to detect unspecific BtuF binding and a fraction of unspecifically bound BtuF was observed. Gel lanes correspond to 1 = initial mix, 2 = flow through, 3 = wash and 4 = elution. MW, marker proteins with masses indicated on the left. (**B**) Amino acid sequence alignment of the six nanobodies selected in pull-down assays, and the non-binder Nb12, which was a member of the second-largest family identified in panning. The three CDR regions are labeled. The secondary structure elements of Nb9 are indicated above the alignment. Cysteine positions for disulfide bond formation are numbered in green. Note the additional disulfide bond between C72 (CDR2) and C128 (CDR3) in Nb9, Nb15 and Nb10. The ESPript server 3.0 available online was used to generate the alignment^48^.

An alpaca was immunized with Cbl-bound BtuF and a nanobody-coding DNA library was prepared from lymphocytes isolated from blood. The library was panned against BtuF using phage display^19,20^, and two rounds of panning were necessary to detect specific enrichment over panning against control solutions. 96 clones of the enriched sub-library were sequenced, which yielded 19 nanobodies with distinct sequences. Five sequences were represented several times and defined as major families. Pull-down assays with immobilized nanobodies and tag-less BtuF identified six BtuF-specific nanobodies: Nb7, Nb9, Nb10, Nb14, Nb15 and Nb17 (Fig. 1A). Whereas Nb7, Nb9 and Nb10 belonged to major families, Nb14, Nb15 and Nb17 were the only nanobodies with this sequence (Fig. 1B).

### Characterization of competitive inhibition of Cbl binding to BtuF by the selected nanobodies

**Figure 2:**
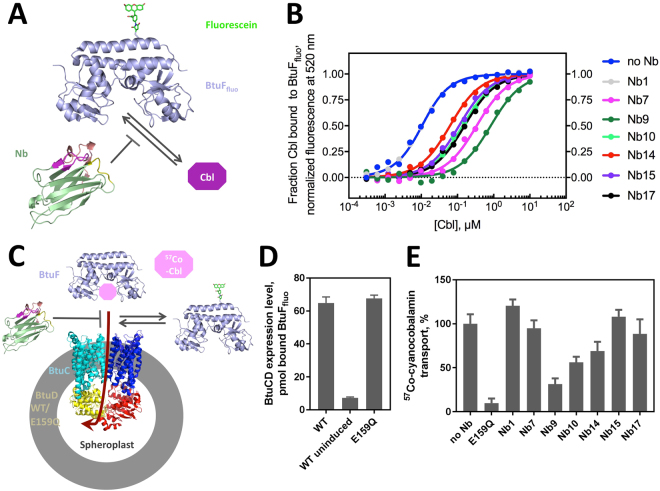
Effect of nanobodies on BtuCD-F function. (**A**) Schematic of the substrate-binding assay. Fluorescently labeled BtuF (BtuF_fluo_) was used to measure cyanocobalamin (Cbl) binding in the presence of nanobody. (**B**) Equilibrium Cbl binding to BtuF_fluo_. Shown is the normalized fluorescence signal against substrate concentration (the raw fluorescence data is shown in Supplementary Figure 1). 5 nM BtuF_fluo_, Cbl concentrations ranging from 0.3 nM to 10 μM, and different Nb concentrations were used (5 μM for Nb1 and Nb14; 1 μM for Nb7, Nb15 and Nb17; 100 nM for Nb9 and Nb10). Affinity values for nanobody-BtuF binding were determined by numerical evaluation of the competitive binding data and shown in Table 1. Note that Nb1 is a control nanobody that does not bind BtuF. **C**) Schematic of the spheroplast-based substrate transport and BtuF_fluo_ binding assays.^57^Co-cyanocobalamin (^57^Co-Cbl) transport into spheroplasts overexpressing WT BtuCD was measured in the presence of Nbs. (**D**) The BtuCD expression level in the spheroplasts was determined by the amount of BtuF_fluo_ associated with the spheroplasts. Cells transformed with a plasmid containing WT BtuCD but without expression induction (‘WT uninduced’) served as a control. The fluorescence was detected using excitation at 485 nm and emission at 516 nm. (**E**) Cbl transport in the presence of Nbs. The following concentrations were used: 5 μM BtuF, 15 μM Cbl, 75 μM nanobodies and 0.08 g/ml spheroplasts (~0.45 μM BtuCD). A hydrolysis-deficient BtuD mutant, E159Q, was used as a negative control. Shown are mean and SEM of the transport rates determined by linear regression using 5 time points.

As a first step to assess the effect of the obtained nanobodies on BtuCD-F function, we determined the affinity of the selected nanobodies to BtuF and their ability to displace Cbl from BtuF. Cbl binding to BtuF was analyzed at pH 7.5 and 23 °C by measuring Cbl-induced fluorescence quenching of fluorescently labeled BtuF (BtuF_fluo_), an assay developed and described previously^6^ (Fig. 2A). Equilibration of BtuF_fluo_ with different Cbl concentrations yielded a dissociation constant (*K*_*d*_) of 8.1 ± 1.0 nM for the BtuF_fluo_-Cbl complex, comparable to the 15 nM determined previously using a radioligand binding assay or isothermal titration calorimetry^13^. We reasoned that the apparent affinity of BtuF_fluo_ for Cbl should decrease at constant concentration of a selected BtuF-specific nanobody if it was a competitive inhibitor of Cbl binding to BtuF. Figure 2B and Table 1 show that all selected, BtuF-specific nanobodies indeed inhibited Cbl binding in a competitive manner (see also Supplementary Figure 1). The nanobodies showed inhibition constants (*K*_*i*_ values) ranging from 770 nM for the weakest binder (Nb14) to 0.94 nM for the binder with highest affinity (Nb9). Two nanobodies (Nb9 and Nb10) thus exhibited a higher affinity for BtuF_fluo_ than its natural ligand Cbl (Table 1). A negative control with a nanobody that does not bind BtuF_fluo_ (Nb1) reproduced the *K*_*d*_ of the BtuF_fluo_-Cbl complex (8.1 nM) within experimental error (Fig. 2B, Table 1), consistent with highly specific BtuF binding by the six selected nanobodies.

**Table 1:** Thermodynamics and kinetics of ligand binding to BtuF_fluo_ at pH 7.5 and 23 °C.

BtuF_fluo_ ligand	*K*_*d*_^1)^ or *K*_*i*_ (M)	*k*_*off*_ (s^−1^)	*k*_*on*_ (M^−1^s^−1^)
Cbl	(8.1 ± 0.3) × 10^−9^ ^1)^	(7.1 ± 0.4) × 10^−1^ ^2)^	(8.8 ± 0.2) × 10^7^ ^3)^
Nb7	(2.4 ± 0.3) × 10^−8^	(1.9 ± 0.01) × 10^−3^	(7.9 ± 0.9) × 10^4^
Nb9	(9.4 ± 1.5) × 10^−10^	(4.2 ± 0.00) × 10^−3^	(4.5 ± 0.7) × 10^6^
Nb10	(6.0 ± 0.3) × 10^−9^	(7.1 ± 0.04) × 10^−3^	(1.2 ± 0.1) × 10^6^
Nb14	(7.7 ± 0.4) × 10^−7^	n.d.	n.d.
Nb15	(7.9 ± 0.6) × 10^−8^	n.d.	n.d.
Nb17	(5.4 ± 0.4) × 10^−8^	(2.5 ± 0.02) × 10^−3^	(4.6 ± 0.3) × 10^4^

**Figure 3:**
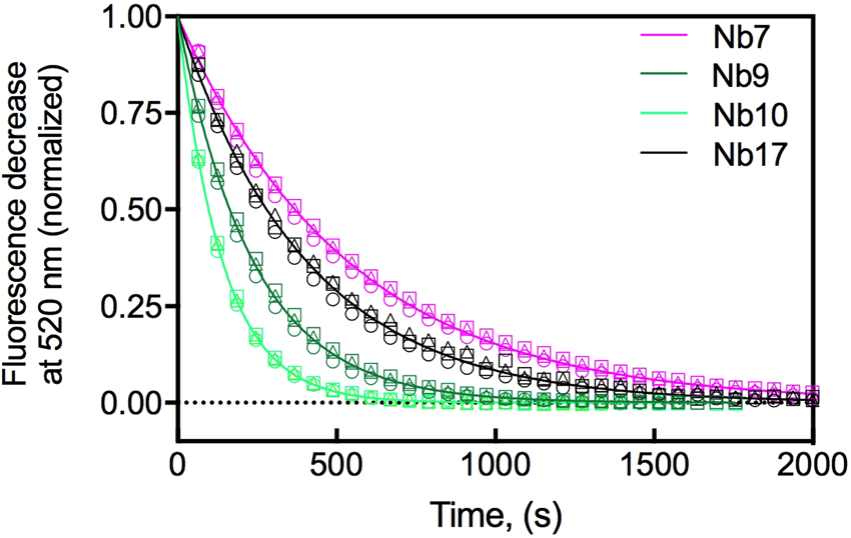
Determination of the rate of spontaneous nanobody dissociation from BtuF_fluo_ (*k*_*off*_) at pH 7.5 and 23 °C. Illustrated are the kinetics of displacement of nanobodies from BtuF_fluo_ by Cbl. Shown is the normalized fluorescence decrease at 520 nm over time (raw data is shown in Supplementary Figure 3). BtuF (10 nM) was incubated with 10 nM nanobody prior to the addition of excess Cbl. The shown fluorescence traces correspond to nanobody displacement by Cbl. Identical rate constants were obtained at three different Cbl concentrations ((○) 1 μM, (∆) 5 μM and (□) 10 μM), showing that the fluorescence decrease directly reported nanobody dissociation from Cbl. The obtained *k*_*off*_ values are listed in Table 1. We also observed a rapid fluorescence decrease during the dead time of manual mixing, presumably caused by the binding of Cbl to free BtuF_fluo_. This rapid phase is not shown for clarity.

To determine the dissociation constants (*K*_*i*_ values) of the BtuF-nanobody complexes, the competitive binding equilibria from Fig. 2B were fitted numerically with *Dynafit* ^43,44^, using the dissociation constant of the BtuF-Cbl complex (8.1 nM) as input and the *K*_*i*_ value of the respective nanbody as open parameter. The dissociation rates (*k*_*off*_) of the BtuF-nanobody complexes were derived from the data in Fig. 3, and the respective association rate constants (*k*_*on*_) were calculated from the equation *k*_*on*_ = *k*_*off*_/*K*_*i*_. The indicated errors correspond to the standard errors derived from the fits. n.d.: not determined.

^1)^Dissociation constant of the BtuFfluo-Cbl complex obtained from equilibrium data (Fig. 2B).

^2)^Calculated from the equation *k*_*off*_ = *K*_*d*_× *k*_*on*_.

^3)^Recorded with stopped-flow fluorescence kinetics (Supplementary Figure 2).

### Effect of purified nanobodies on Cbl import into spheroblasts *in vitro*

*In vitro* ^57^Co-cyanocobalamin transport was measured with spheroplasts prepared from *E. coli* cells containing over-expressed wild-type (WT) BtuCD (Fig. 2C). A hydrolysis-deficient mutant, BtuCD_E159Q_, was used as a negative control. Similar BtuCD expression levels were measured in spheroplasts with WT BtuCD or BtuCD_E159Q_, as determined by the amount of BtuF_fluo_ that was associated with the spheroplasts (Fig. 2D). Cbl transport was reduced to 30% in the presence of Nb9, but a 5-fold molar excess of nanobody over Cbl was required (Fig. 2E). A 2-fold molar excess of Nb9 over Cbl resulted in 50% remaining activity (data not shown). Reduction of transport was also detected for Nb10 (to 50%) and Nb14 (to 70%) compared to the uninhibited rate. Nb7, Nb15 and Nb17, however, hardly affected substrate transport even at high nanobody concentrations.

### Kinetics of binding and dissociation of nanobody–BtuF complexes

As nanobody binding to BtuF_fluo_ did not cause significant fluorescence changes in BtuF_fluo_, we set up a competitive ligand displacement experiment that allowed determination of the rate of spontaneous dissociation of the selected nanobodies from BtuF_fluo_ (*k*_*off*_) as well as calculation of association rates (*k*_*on*_) from the respective *k*_*off*_/*K*_*i*_ ratios. BtuF_fluo_ (10 nM) was first incubated with an equimolar amount of nanobody. The resulting solution containing free BtuF_fluo_ and BtuF_fluo_-nanobody complex was then mixed with excess Cbl (1, 5, or 10 μM) and the decrease in BtuF_fluo_ fluorescence at 520 nm was recorded. The biphasic kinetics were characterized by a very rapid fluorescence decrease caused by Cbl binding to free BtuF_fluo_ (completed within the dead time of manual mixing) and a slow phase during which Cbl bound to BtuF_fluo_ that had dissociated from the respective nanobody. All slow phases proved to be independent of excess Cbl concentration, demonstrating that the slow phase of fluorescence decrease directly monitored nanobody dissociation from BtuF_fluo_ (Fig. 3). The *k*_*on*_ and *k*_*off*_ values thus determined for the four nanobodies with highest affinity to BtuF (Nb7, Nb9, Nb10, and Nb17) are summarized in Table 1. The results showed that all nanobodies exhibited similar, slow off-rates ranging from 1.9‒7.1 × 10^−3^ s^−1^ (corresponding to dissociation half-lives of 1.6‒6.1 minutes) and that differences in their *K*_*i*_ values mainly resulted from large differences in their association rates with BtuF (4.6 × 10^4^ M^−1^s^−1^‒4.5 × 10^6^ M^−1^s^−1^). Nb9, the highest affinity binder of BtuF, showed the highest on-rate (4.5 × 10^6^ M^−1^s^−1^), but still associated 20 times slower with BtuF_fluo_ than Cbl (Table 1, Supplementary Figures 2 and 3). In addition, the fact that all nanobodies dissociated about two orders of magnitude slower from BtuF_fluo_ than Cbl (Table 1) suggest that the nanobodies were selected for slow dissociation from BtuF during phage display.

### Crystal structure of the Nb9-BtuF complex

**Figure 4:**
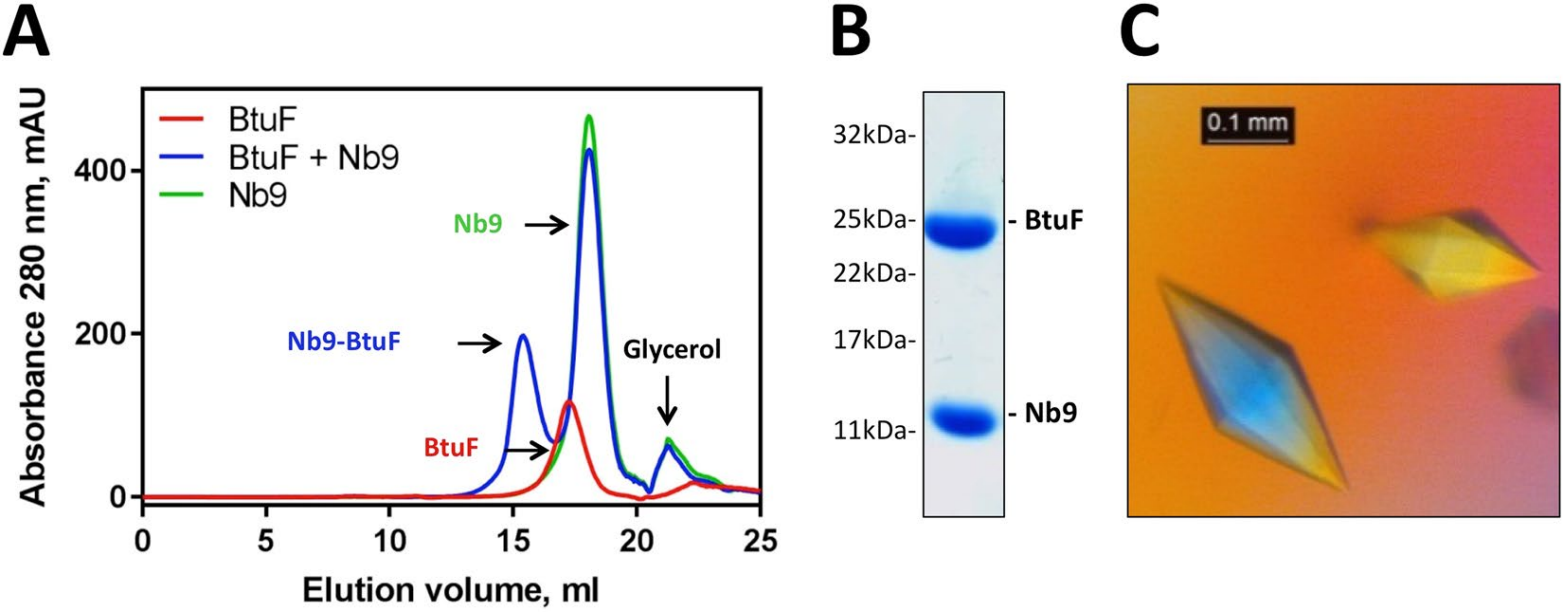
Purification and crystallization of Nb9-bound BtuF. (**A**) Size exclusion chromatography of the Nb9-BtuF complex. Note the shift of the elution peak of the Nb9-BtuF complex compared to the individual proteins. Equivalent amounts of protein (710 μg BtuF, 3.2 mg Nb9) were injected in all experiments. (**B**) SDS-PAGE analysis of isolated Nb9-BtuF complex after gel filtration confirmed the presence of both components. (**C**) Crystals of the Nb9-BtuF complex grown in optimized screens containing 100 mM Tris-HCl pH 8.5, 400 mM MgCl_2_ and 33% w/v PEG4000.

**Figure 5:**
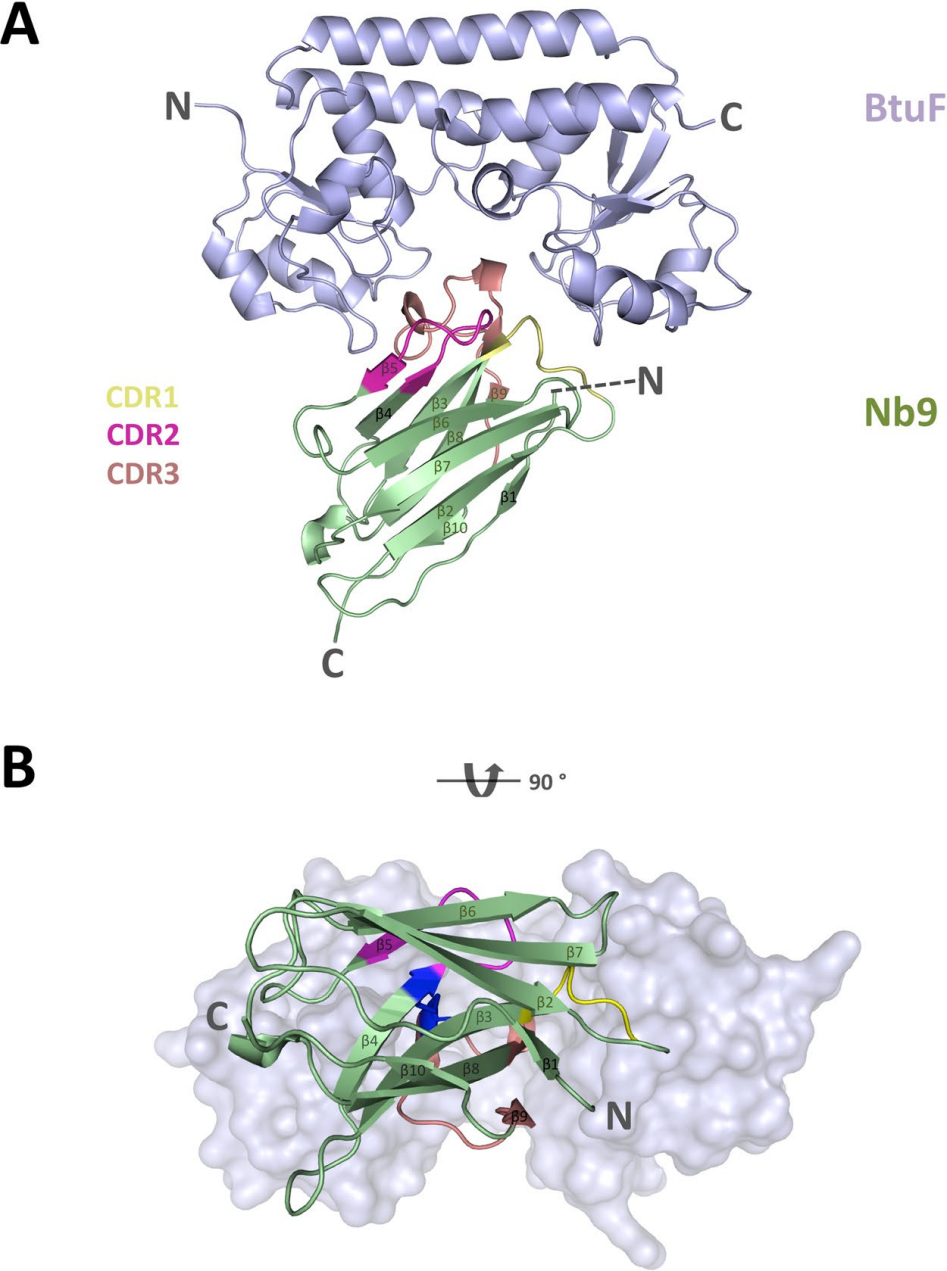
Crystal structure of the Nb9-BtuF complex. (**A**) Ribbon diagram of BtuF in light blue, and Nb9 colored green with CDR1, CDR2 and CDR3 regions colored yellow, magenta and salmon, respectively. The amino- and carboxy-termini are indicated with N and C, respectively. (**B**) View onto the substrate-binding and Nb-binding site. Note the stabilizing disulfide bond in dark blue between the CDR2 (C72) and CDR3 (C128) loops. BtuF is displayed in light blue surface representation.

**Figure 6:**
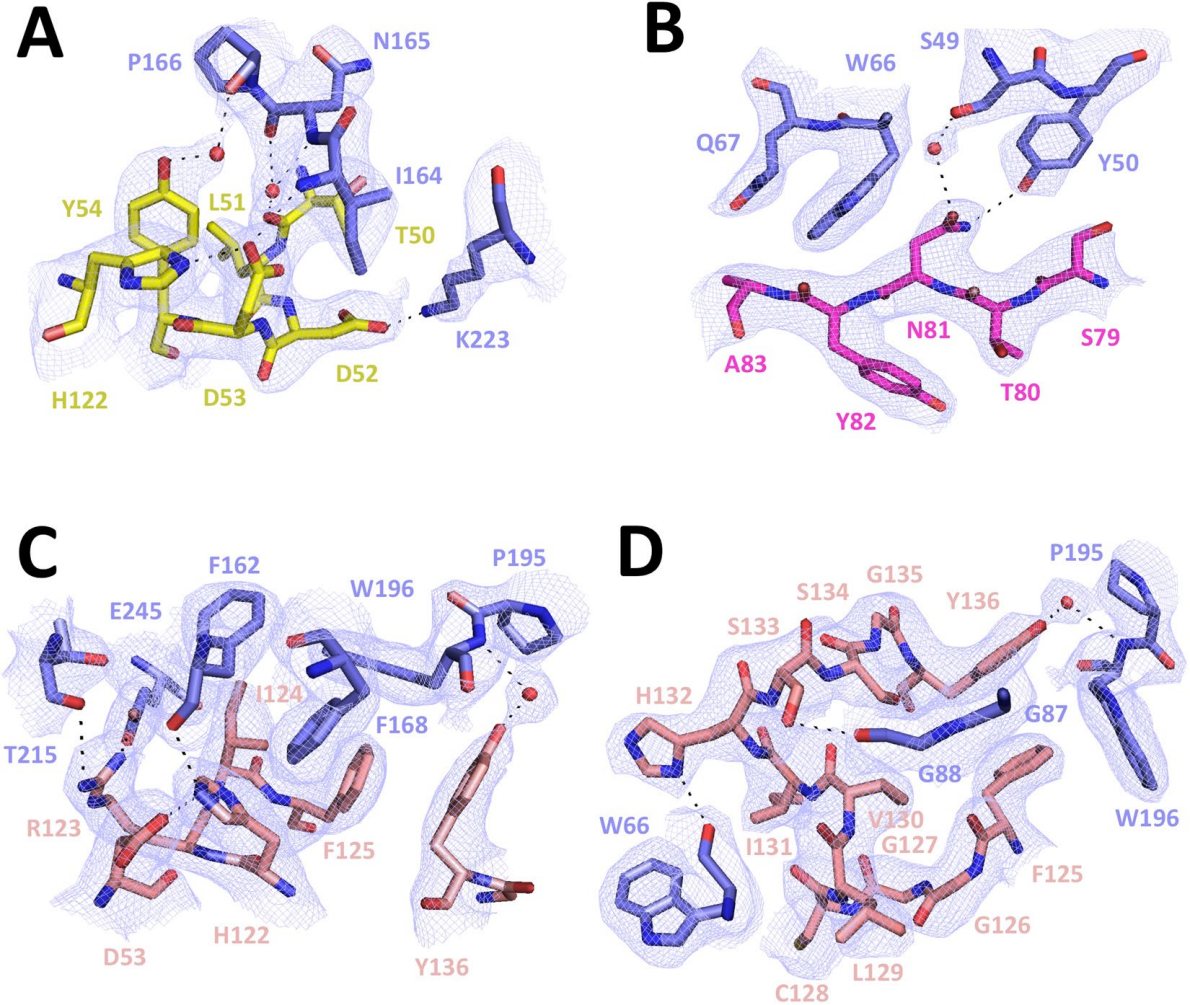
Details of the Nb9-BtuF interface. Illustrated are the interactions of BtuF with CDR1 (**A**), CDR2 (**B**), CDR3 N-terminal part (**C**) and CDR3 C-terminal part (**D**). BtuF is shown in blue and the CDR regions are colored yellow (CDR1), magenta (CDR2) and salmon (CDR3). Oxygen and nitrogen are shown in red and blue. Water molecules are indicated with red spheres. Shown is the sigma A-weighed 2Fo-Fc electron density map contoured at 1σ.

To visualize the structural basis of the observed inhibitory effects of Nb9, we determined the crystal structure of the Nb9-BtuF complex. The complex was purified by size exclusion chromatography, and SDS-PAGE analysis confirmed the presence of both components (Fig. 4). The crystals showed anisotropic data and the structure of the Nb9-BtuF complex was refined to 2.7 Å resolution (Table 2). The nanobody bound to the Cbl-binding pocket of BtuF, where BtuC-binding would occur (Fig. 5). Mainly polar and electrostatic interactions were present at the Nb9-BtuF interface. The CDR3 loop inserted approximately 10 Å into the Cbl-binding pocket and several BtuF residues involved in Cbl binding (Y50, W66, F162, F168 and W196) were contributing to nanobody binding. Extensive hydrogen bonding was found between the side chains of CDR3 and the backbone of BtuF, and a salt bridge was formed by R123 and E245A in BtuF (Fig. 6). A hydrophobic patch and aromatic stacking were observed involving CDR3 residues I124, F125, Y136 and BtuF residues F162, F168 and W196. The side chain residues of CDR1 were engaged in direct and indirect hydrogen bonds with the backbone of BtuF, and D52 formed a salt bridge with K223 in BtuF. In CDR2, N81 was involved in direct or water-mediated hydrogen bonding with the side chains of Y50 and S49.

**Table 2:** Data processing and refinement statistics.

	Nb9-BtuF
**Data collection**	
Wavelength (Å)	0.99984
Resolution range (Å)	19.92–2.653 (2.748–2.653)
Space group	P 21 21 21
Unit cell dimensions	
a, b, c (Å)	83.8 141 216.7
α, β, γ (°)	90 90 90
Total reflections	508253 (49591)/[432073 (5531)]
Unique reflections	75414 (7453)/[63917 (957)]
Multiplicity	6.7 (6.7)/[6.8 (5.8)]
Completeness (%)	99.63 (99.95)/[85.08 (12.94)]
Mean I/sigma(I)	14.96 (1.47)/[17.14 (4.18)]
Wilson B-factor (Å^2^)	61.48/[47.57]
*R*_*merge*_ (%)	11.5 (152.6)/[9.8 (47.0)]
*R*_*meas*_ (%)	12.5 (165.5)/[10.6 (51.5)]
CC _½_ (%)	99.9 (61.8)/[99.9 (90.7)]
**Refinement**	
Reflections used in refinement	63907 (957)
Reflections used for R-free	3239 (50)
*R*_*work*_ (%)	22.7 (38.2)
*R*_*free*_ *(5% of data)* (%)	26.2 (37.4)
No of non-hydrogen atoms	17355
Macromolecules	17058
Ligands	30
Water	264
Protein residues	2226
RMS bonds (Å)	0.003
RMS angles (°)	0.56
Ramachandran favored (%)	95.05
Ramachandran allowed (%)	4.41
Ramachandran outliers (%)	0.54
Rotamer outliers (%)	1.04
Clashscore	5.94
Average B-factor (Å^2^)	102
Macromolecules	103
Ligands	91.0
Solvent	47.0
Number of TLS groups	18

Highest-resolution shell values are shown in parentheses and numbers in brackets are after ellipsoidal truncation and anisotropic correction. The correlation coefficient is abbreviated CC^49^.

### Comparison of the Nb9-BtuF complex to Cbl-bound BtuF

**Figure 7:**
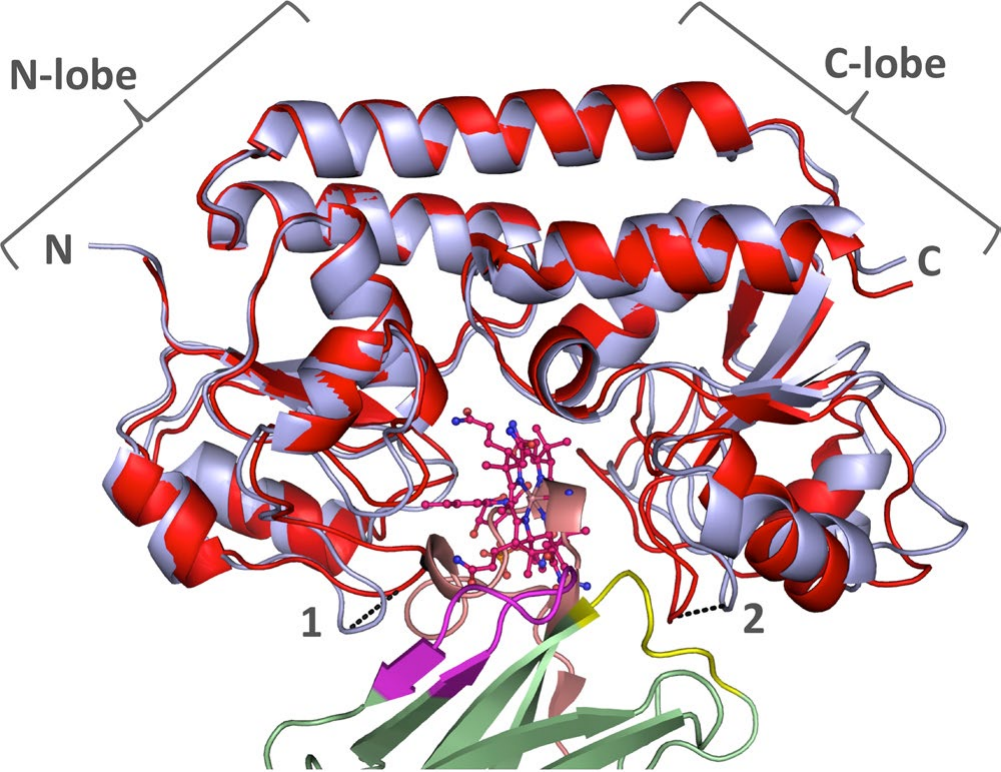
Comparison of Nb9-BtuF complex with Cbl-bound BtuF. Nanobody-bound BtuF is shown in light blue cartoon representation, with the nanobody in green with colored CDRs (CDR1, yellow; CDR2, magenta; CDR3, salmon). Cbl-bound BtuF (PDB ID 1N4A) is displayed in red and Cbl is shown in stick representation with oxygen in red, nitrogen in blue, and cobalt in brown. The two BtuF chains superimposed with an RMSD of 1.53 Å over 243 atoms. Distances of Cα position between corresponding atoms in the loops gating the entrance of the substrate-binding pocket are indicated (1: 5.5 Å, Q67 in Nb9-BtuF and 2: 4.4 Å, N165 in Nb9-BtuF). The C-lobe of BtuF displays more pronounced structural changes than the N-lobe.

Nb9 shared an interface of 1054 Å^2^ with BtuF, which was larger than that of BtuF with Cbl, 786 Å^2^, but smaller than the BtuF-BtuC_2_ interface in the BtuCD-F complex, which amounts to 2612.5 Å^2^ (PDBe PISA v1.51 [22/09/2014]). The Nb9-BtuF complex superimposed to Cbl-bound BtuF (PDB ID 1N4A) with an RMSD of 1.53 over 243 atoms (Fig. 7). Comparing the two structures, a 9.9 Å opening was observed between the loops gating the entrance of the substrate binding pocket in Nb9-bound BtuF. Additional structural differences induced by nanobody binding were also observed in the C-lobe of BtuF.

## Discussion

We investigated the functional inhibition of an ABC importer by targeting its cognate SBP with a nanobody. The alpaca had been injected with Cbl-bound BtuF, but Nb-BtuF binding in the pull-down assay did not contain or require Cbl, and all generated nanobodies were found to compete with Cbl for BtuF binding. The structure of the Nb9-BtuF complex confirmed that Cbl must have been displaced from the binding site during the selection of this nanobody. Presumably, BtuF lost its bound Cbl to high affinity Cbl binders like transcobalamin present in the alpaca blood^21^. The Nb9-BtuF crystal structure revealed that Nb9 bound to BtuF by inserting CDR3 into the deep Cbl-binding cleft. The recognition of conformational motifs, in particular clefts, is typical for nanobodies and has previously been reported for other proteins^22^, including enzymes that were found to be inhibited if CDRs of nanobodies were inserted into active sites^23,24^.

Of the six BtuF-specific nanobodies with inhibitory potential, Nb9 showed the strongest effects and was the highest affinity binder. The dissociation constant was determined to be 1 nM, revealing an affinity that is higher than most nanobody-antigen affinities determined by surface plasmon resonance^25,26^. It was even higher than the affinity of BtuF for Cbl (its substrate), which explained the strong inhibitory effect of Nb9 in the substrate-binding assays (an equilibrium reaction), even at low nanobody concentration.

In contrast, a high nanobody concentration was required to observe inhibition of substrate transport (a non-equilibrium reaction). Under the experimental conditions used (and considering only the Nb, BtuF and Cbl concentrations and affinity constants), only 2.5% remaining transport activity was expected in the presence of Nb9. However, 30% remaining activity was measured. There are at least three possible explanations for this apparent discrepancy: First, the fact that Nb9 association with BtuF_fluo_ is 20 times slower compared to Cbl binding results in faster binding of the small substrate compared to the bigger nanobody. Second, in the transport reaction, the nanobody not only competes with Cbl for binding to BtuF, but also with BtuCD-F complex formation. The interaction of BtuF with BtuCD is an essential component of the transport reaction, and the dissociation constant of the complex has been reported to be between nanomolar and picomolar^18,27^. Third, because of the high affinity interaction with BtuCD, it has earlier been speculated that BtuF may not fully dissociate from BtuCD during the transport cycle^28^. Partial displacement may be sufficient for the binding of another Cbl molecule, but not for nanobody binding. The nanobody would therefore be excluded from binding BtuF partially dissociated from BtuCD as long as sufficient Cbl is available to be transported. These three scenarios can rationalize why *in vitro* transport inhibition was only observable for certain nanobodies and when used at high concentrations. Nevertheless, the nanobodies may be more efficient when substrate is limiting, given that SBPs such as BtuF will often be in substrate-free state. While this would inhibit a transporter *in vivo*, it would not allow detection *in vitro* given the slow transport rates of type II ABC importers including BtuCD-F.

The crystal structure of the Nb9-BtuF complex provided the structural basis of the observed functional effects. Due to steric hindrance, Nb9 binding to BtuF was incompatible with simultaneous binding of Cbl or BtuCD. Since the other nanobodies also competed with Cbl binding, they presumably bind BtuF in a similar way. Nb9 might have an overlapping BtuF binding site with Nb15, since they share similarities in their CDRs. All the CDR3 residues of Nb9 involved in BtuF binding were conserved in Nb15, except for F125. This residue was engaged in aromatic stacking in a hydrophobic patch and is replaced by a leucine in Nb15. This substitution retains the hydrophobicity, but the aromatic stacking property is lost. All CDR1 residues involved in BtuF binding were conserved in Nb15. In CDR2, residue N81 of Nb9 was substituted with a histidine in Nb15, maintaining the hydrogen bonding properties. Even though Nb9 and Nb15 probably share the same binding site, the strength of their interaction with BtuF is different. The structural data provide a plausible explanation why the substitutions in CDR2 and CDR3 of Nb15 are responsible for an about 80-fold decrease in BtuF affinity compared to the one of Nb9.

Nanobodies have been used for several purposes other than functional inhibition. They were successfully used as crystallization chaperones because of their ability to rigidify flexible regions, to mediate crystal contacts or to stabilize conformational states of enzymes and transporters^29,30^. Nanobody-based products like chromobodies (Nb coupled to a fluorescent protein, i.e. GFP) are also helpful diagnostic tools to image the localization of the target protein within the cells or quantify expression^31–33^. In the field of drug discovery, nanobody-drug-conjugates have a potential as clinical therapeutics, because they bind specifically to their target and are less immunogenic than conventional antibodies^34–36^. The herein described approach to inhibit the uptake of essential nutrients by targeting the SBP with a nanobody might have a value for targeting pathogenic bacteria, thus offering a novel antibiotic strategy. The pathogenesis of certain organisms has been associated with the function of ABC importers for nutrient acquisition and growth. Potential targets are the *Staphylococcus aureus* heme importer or the *Streptococcus pyogenes* maltodextrin importer. Both are Gram-positive organisms in which the import system is directly accessible for the nanobody, without the necessity to cross an outer membrane^37,38^. Selected nanobodies are highly specific for the target system and initial binders will likely require affinity maturation, which can be performed in a rational way if crystal structures of the nanobody-target complex such as the Nb9-BtuF structure are available^39^.

## Materials and Methods

### BtuF purification

BtuF for the pull-down, crystallization, binding and transport assays, was prepared as described previously^6,18^. The Avi-tagged BtuF construct for the panning was cloned by insertion of a C-terminal Avi-tag between the BtuF gene and the 3 C cleavable His-tag. The construct was expressed and purified as described previously^6^. Biotinylation was performed according to manufacturer’s instructions (Avidity, protocol for BirA enzyme). BirA was added to BtuF in a 1:2 mass ratio and the buffer contained 10 mM MgAcetate, 10 mM ATP, 50 μM Biotin, 10 mM Tris-HCl pH 8 and 100 mM NaCl. The biotinylation reaction was carried out overnight at 4 °C. His-tagged BirA was removed by IMAC. The sample was purified by size exclusion chromatography with a superdex 200 column. The final buffer was composed of 10 mM Tris-HCl pH 8, 100 mM NaCl and 10% v/v glycerol. Biotinylated BtuF was snap frozen in liquid nitrogen and stored at −80 °C.

### Generation of nanobodies against BtuF

Nanobodies were produced following a modified version of the protocols established in the Steyaert and Dutzler laboratories^19,20^. In brief, an alpaca was injected with 200 – 300 μg of Cbl-bound BtuF for the generation of heavy chain only antibodies. Four injections were performed each in an interval of two weeks, and the specific immune response was analyzed by ELISA. 1½ weeks after the last injection, 50 ml blood was collected for subsequent lymphocyte preparation with Histopaque-1077 (Sigma 10771) and ACCUSPIN tubes (Sigma A2055). Isolation of the total RNA was performed with the RNeasy Mini Kit according to manufacturer’s procedure (Qiagen, 74104). The cDNA was prepared by reverse transcription and the antibody fragments were amplified by PCR using primers described previously^40,41^. The PCR products corresponding to the fragment derived from the heavy chain only antibody were isolated by preparative agarose gel and purified with a Gel Extraction Kit (Qiagen 28706). The variable domain of the heavy chain only antibody (VHH) sequences were further amplified by a second PCR and FX cloning sites were introduced to insert the VHH DNA into the phagemid vector for phage display^42^. Electrocompetent TG1 cells were transformed with the VHH DNA library for storage and further preparation of the phage particles. For phage display, biotinylated BtuF was immobilized on a neutravidin coated plate (Thermo Scientific, 15507) and omission of protein or immobilized transcobalamin were used as controls to quantify enrichment. The phage library was panned against apo- and Cbl-bound BtuF by phage display, and two rounds of panning were necessary to detect enrichment. A 96-well plate with individual clones of the enriched sub-libraries was sent for DNA sequencing.

### Expression and purification of nanobodies

*E. coli* WK6 cells were transformed with plasmids purified from TG1 cells by standard MiniPrep procedure. Expression cultures were inoculated with a pre-culture, and cells were grown at 37 °C and 190 rpm in Terrific Broth (TB) medium containing 100 μg/ml ampicillin, 1% (w/v) glucose and 2 mM MgCl_2_ until an OD_600_ of ~0.7 was reached. The temperature was lowered to 25 °C and protein expression was induced with 1 mM IPTG overnight. Cells were harvested by centrifugation, and the nanobodies were extracted from the periplasm. Therefore, cells were resuspended in TES buffer composed of 0.2 M Tris-HCl pH 8, 0.5 mM EDTA and 0.5 M sucrose, and incubated on a rotating wheel for 1 hour at 4 °C. The cell suspension was diluted with double the volume of TES/4, incubated for another hour at 4 °C and centrifuged to isolate the supernatant containing the periplasmic extract. His-tagged nanobodies were purified by IMAC and desalted with PD10 columns following the manufacturer’s instructions (GE healthcare). The storage buffer contained 250 mM NaCl, 50 mM Tris-HCl pH 7.5, 0.5 mM EDTA and 10% v/v glycerol.

### Pull-down assay

His-tagged nanobodies were immobilized on Ni-NTA and incubated with 1.5 molar excess of tagless BtuF for 1.5 hours at 4 °C. Bound protein was separated from excess BtuF by a quick spin and washed with 20 column volumes of wash buffer composed of 50 mM Tris-HCl pH 7.5, 250 mM NaCl, 10% v/v glycerol and 20 mM imidazole-HCl pH 8. Bound protein was eluted with 500 mM imidazole-HCl pH 8 and analyzed by SDS-PAGE.

### Affinity determination of BtuF_fluo_ for Cbl and the selected nanobodies

A fluorescein-labeled variant of BtuF (BtuF_fluo_) that shows an about 4-fold decrease in fluorescence at 520 nm upon Cbl binding was used to monitor the BtuF/Cbl interaction in solution, as described previously^6^. For the determination of the dissociation constant of the BtuF_fluo_-Cbl complex, BtuF_fluo_ (5 nM) was incubated for 1 hour at 23 °C with different Cbl concentrations (0.3 nM to 10 μM) in 50 mM Tris-HCl pH 7.5, 200 mM NaCl, 0.001% (v/v) Tween 20. The fluorescence at 520 nm in each sample was recorded for 10 s on a QM 7/2003 fluorescence spectrometer (PTI) (excitation at 495 nm, slit 3 nm; emission at 520 nm, slit 12 nm) and averaged. Fluorescence intensities were plotted against Cbl concentration and fitted according to a simple non-covalent binding equilibrium with Origin (OriginLab, Northhampton, USA).

For the determination of dissociation constants (*Ki* values) of BtuF-nanobody complexes, equilibrium competition experiments were performed with displacement of Cbl from BtuF_fluo_ by the respective nanobody as readout. BtuF (5 nM) was incubated with different Cbl concentrations as described above, but at a constant concentration of the respective nanobody (5 μM for Nb1 and Nb14; 1 μM for Nb7, Nb15 and Nb17; 100 nM for Nb9 and Nb10). The fluorescence of BtuF_fluo_ was plotted against Cbl concentration, and the data were normalized and fitted with *Dynafit* ^43,44^ according to a competitive binding equilibrium with mutually exclusive binding of Cbl and nanobody to BtuF_fluo_, with the *K*_*d*_ of Cbl for BtuF (8.1 nM) as input and the *K*_*i*_ of the nanobody-BtuF complex as open parameter.

### Kinetics of nanobody binding to BtuF

Dissociation rates of nanobodies were determined by measuring the kinetics of nanobody displacement from BtuF_fluo_ using Cbl. BtuF_fluo_ (10 nM) was equilibrated with equimolar amounts of nanobody at 23 °C in 50 mM Tris-HCl pH 7.5, 200 mM NaCl, and 0.001% (v/v) Tween 20. Depending on the binding affinity, an occupancy of 72% (Nb9), 53% (Nb10), 30% (Nb7) or 14% (Nb17) of BtuF_fluo_ with nanobody was achieved. After addition of Cbl to final concentrations of 1 μM, 5 μM or 10 μM, the kinetics of fluorescence decrease at 520 nm were recorded (excitation at 495 nm, slit: 2 nm; emission slit: 10 nm). A rapid fluorescence decrease (binding of Cbl to free BtuF_fluo_) was followed by a slow phase of fluorescence decrease that corresponded to the dissociation of nanobody-BtuF_fluo_ complexes because it proved to be independent of the Cbl concentration used. The data of the slow phase were fitted according to first-order kinetics and yielded the dissociation rates (*k*_*off*_) of the nanobody-BtuF_fluo_ complexes.

### Stopped-flow fluorescence kinetics of Cbl binding to BtuF_fluo_

The kinetics of Cbl binding to BtuF_fluo_ were recorded under preudo-first-order conditions via the fluorescence decrease above 520 nm using a SX20 stopped-flow spectrometer (Applied photophysics). Reactions were performed in 50 mM Tris-HCl pH 7.5, 150 mM NaCl, 0.0001% Tween 20 at 23 °C and initiated by mixing at a 1:1 ratio. The final concentration of BtuF_fluo_ was 10 nM in all experiments, and the final Cbl concentrations varied between 39 nM and 625 nM. The observed first-order rate constants (*k*_*obs*_) were plotted against the Cbl concentration, and the association rate of Cbl binding to BtuF_fluo_ (*k*_*on*_) was determined from the slope of the *k*_*obs*_ vs. [Cbl] plot.

### Spheroplast preparation

*E. coli* BL21 (DE3) Gold cells were transformed with plasmids encoding cysteine free wild-type BtuCD or a hydrolysis-deficient mutant BtuCD_E159Q_. Cells were grown in TB medium supplemented with 1% (w/v) glucose and 100 μg/ml Ampicillin until the culture reached OD_600_ of 1. Protein expression was induced with 0.25 mM IPTG for 1 hour at 37 °C. Cells were harvested by centrifugation and the pellet was stored at −80 °C. To prepare spheroplasts^45,46^, about 0.2 g cell pellet was washed with 10 ml of 0.2 M Tris-HCl pH 8 and finally resuspended in 2 ml. An equal volume of 1 M sucrose in 0.2 M Tris-HCl pH 8 was added, followed by addition of lysozyme and EDTA to final concentrations of 50 μg/ml and 0.5 mM. Samples were diluted with an equal volume of cold ddH_2_0 and supplemented with MgCl_2_ and DNase I to final concentration of 3 mM and 20 μg/ml. To remove buffer components, the spheroplasts were pelleted by centrifugation and resuspended at 0.16 g/ml in 0.25 M sucrose, 0.05 M Tris-HCl pH 8 and 3 mM MgCl_2_. All procedures were performed on ice and spins were done at 4 °C.

### BtuCD concentration in spheroplasts

To determine the amount of functionally expressed BtuCD in the spheroplasts, 4 μM BtuF_fluo_ was incubated with an equal volume of 0.16 g/ml spheroplasts for 30 min at RT. Spheroplasts were pelleted to remove unbound excess BtuF_fluo_. The pellet was washed with resuspension buffer containing 0.25 M sucrose, 0.05 M Tris-HCl pH 8 and 3 mM MgCl_2_. Spheroplasts with bound BtuF_fluo_ were resuspended at 0.08 g/ml spheroplasts and dissolved with 1.7% SDS. The fluorescence associated with the sample was measured with a plate reader using excitation at 485 nm and emission at 516 nm. The amount of bound BtuF_fluo_ was determined by using a BtuF_fluo_ calibration curve.

### Substrate transport assay with spheroplasts

Spheroplasts and BtuF preincubated with nanobodies and cyanocobalamin for 1 hour were equilibrated to RT. 5 μM BtuF, 75 μM nanobodies, 15 μM cyanocobalamin (^57^Co-Cbl, MP Biomedicals 06B-430000, 10.5 μCi) and 0.08 g/ml spheroplasts (~0.45 μM BtuCD) were used for the reaction and the buffer was composed of 0.25 M sucrose, 0.05 M Tris-HCl pH 8 and 3 mM MgCl_2_. Time points were taken by diluting 50 μl of the reaction in 300 μl ice cold stop buffer composed of reaction buffer supplemented with 8% w/v PEG6000 and 100 μM cold Cbl. The stopped reaction mix was transferred to a manifold filtration system (Millipore MSFBN6B) and washed two times with 200 μl cold stop buffer. The trapped radioactivity on the filters was measured with a γ counter and initial transport rates were determined by linear regression in Graphpad Prism. The transport rates were corrected for BtuCD concentration in the spheroplasts.

### Purification and crystallization of the Nb9-BtuF complex

Purified, tagless BtuF was mixed with Nb9 using a 1.3 - fold molar excess of nanobody. The complex was isolated by size exclusion chromatography using a superdex 200 column equilibrated with 10 mM Tris-HCl pH 7.5 and 100 mM NaCl. The sample was concentrated to approximately 20 mg/ml and 17% excess nanobody was added to the final sample. Crystallization experiments were performed at 20 °C with home-made and commercial screens using 1:1 and 2:1 drop ratios. Crystals grew in various conditions and initial hits of the best diffracting crystals were obtained in JCSGIV B9 (100 mM Tris-HCl pH 8.5, 200 mM MgCl_2_, 30% w/v PEG4000) in a 1:1 drop ratio. Transparent bipyramidal crystals appeared after 2 days and grew to full size (~250 microns) within a few days. For data collection, crystals were cryoprotected with 25% v/v glycerol.

### Structure determination of the Nb9-BtuF complex

Data collection was performed at the Swiss Light Source at PSI Villigen. Data processing was done with the XDS package. The structure of Nb9-bound BtuF was determined by molecular replacement in Phenix using apo-BtuF (PDB ID 1N4D) and a nanobody scaffold (cleaved CDRs) as search models. The phasing process was not straight forward, and a stepwise process using partial solutions from previous runs had to be performed iteratively until all chains in the asymmetric unit were found. Two nanobody chains had to be placed in manually because of poor electron density for automated phasing. Six copies of the Nb-BtuF complex, equivalent to 12 protein chains in total, were present in the asymmetric unit (Supplementary Figure 4). Therefore, strict NCS was used in refinement throughout the structure determination process. Chains D and J displayed the best electron density for BtuF and Nb9, respectively, and thus used for model building. For nanobody chain I, only ~50% of the protein chain was visible in the electron density and represented by high B factors for many residues. Ellipsoidal truncation and anisotropic correction were applied to the data using the diffraction anisotropy server (UCLA-DOE LAB, Supplementary Figure 5)^47^.

## Electronic supplementary material

Supplementary Information
