# Supplementary material for: Structural basis of nanobody-mediated blocking of BtuF, the cognate substrate-binding protein of the Escherichia coli vitamin B12 transporter BtuCD
Source: Sci Rep. 2017 Oct 30;7:14296. doi: 10.1038/s41598-017-14512-8 (PMC5662663; doi:10.1038/s41598-017-14512-8)
Supplement: Supplementary file 1 — Supplementary Information [file 41598_2017_14512_MOESM1_ESM.pdf]

Structural basis of nanobody-mediated blocking of BtuF, the cognate substrate-binding protein of the *Escherichia coli* vitamin B12 transporter BtuCD

Supplementary information

Mireku S.A., Sauer M.M., Glockshuber R. and Locher K.P.

Institute of Molecular Biology and Biophysics, Eidgenössische Technische Hochschule (ETH) Zürich, CH-8093 Zürich, Switzerland

Corresponding author: Kaspar Locher, +41-44-633-3991, locher@mol.biol.ethz.ch

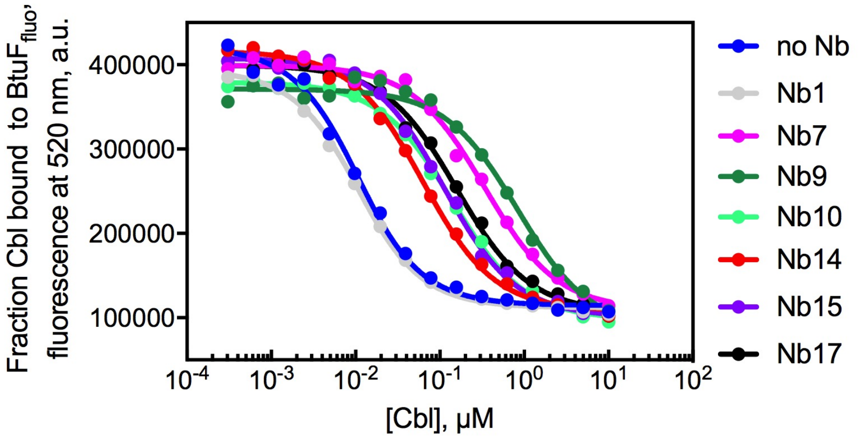

Supplementary Figure 1: Equilibrium Cbl binding to BtuF$_{fluo}$ in the absence or presence of nanobodies. Shown is the raw fluorescence data of the competitive binding assay presented in Figure 2B prior to normalization. The fluorescence at 520 nm of BtuF$_{fluo}$ (5 nM in all experiments) at pH 7.5 and 23°C was recorded as a function of Cbl concentration, either in the absence of nanobody or in the presence of a constant concentration of a specific nanobody (5 µM for Nb1 and Nb14; 1 µM for Nb7, Nb15 and Nb17; 100 nM for Nb9 and Nb10). Note that Nb1 is a control nanobody that does not bind BtuF. All nanobodies were used at concentrations above (6.5 to 106-fold) their inhibition constants (see Table 1), establishing nearly complete occupancy of BtuF$_{fluo}$ with the respective nanobody prior to addition of Cbl. The data were fitted according to a competition between nanobodies and Cbl for binding to BtuF$_{fluo}$, with the initial and final fluorescence signal and the inhibition constants (K_i values) of the individual nanobodies (Table 1) as open parameters (solid lines) and the dissociation constant of BtuF$_{fluo}$-Cbl complex (8.1 nM) obtained from the data in the absence of nanobody as fixed parameter. Note that the final fluorescence values (fluorescence of the BtuF-Cbl complex) are identical in all equilibrium titrations.

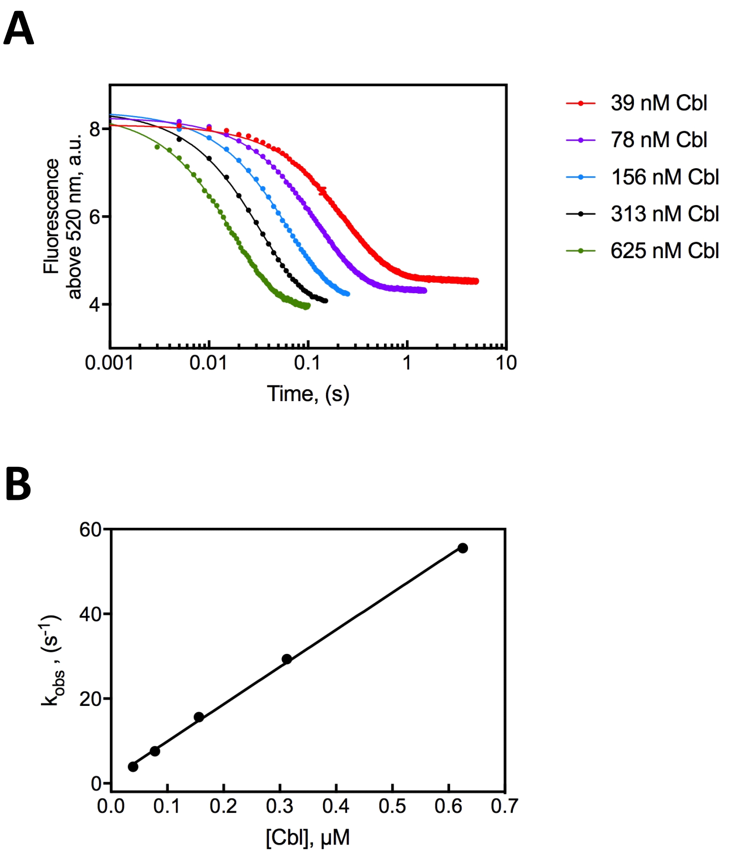

Supplementary Figure 2: Stopped-flow fluorescence kinetics of Cbl binding to $BtuF_{fluo}$ at pH 7.5 and 23 °C. (A) Original fluorescence traces (fluorescence above 520 nM) recorded after mixing $BtuF_{fluo}$ (final concentration: 10 nM) with different amounts of Cbl (final concentrations: 39 nM, 78 nM, 156 nM, 313 nM and 625 nM). Data were fitted according to first-order kinetics (solid lines). (**B**) Observed first-order rate constants (k_{obs}) determined in **A** plotted against Cbl concentration. The slope of the linear fit corresponds to k_{on} and yielded a value of 8.8±0.2 x 10^7 $M^{-1}s^{-1}$ (see Table 1). The rate of spontaneous dissociation of the $BtuF_{fluo}$-Cbl complex (k_{off}) was calculated from the product of k_{on} and the K_d value determined in Figure 2B (see Table 1).

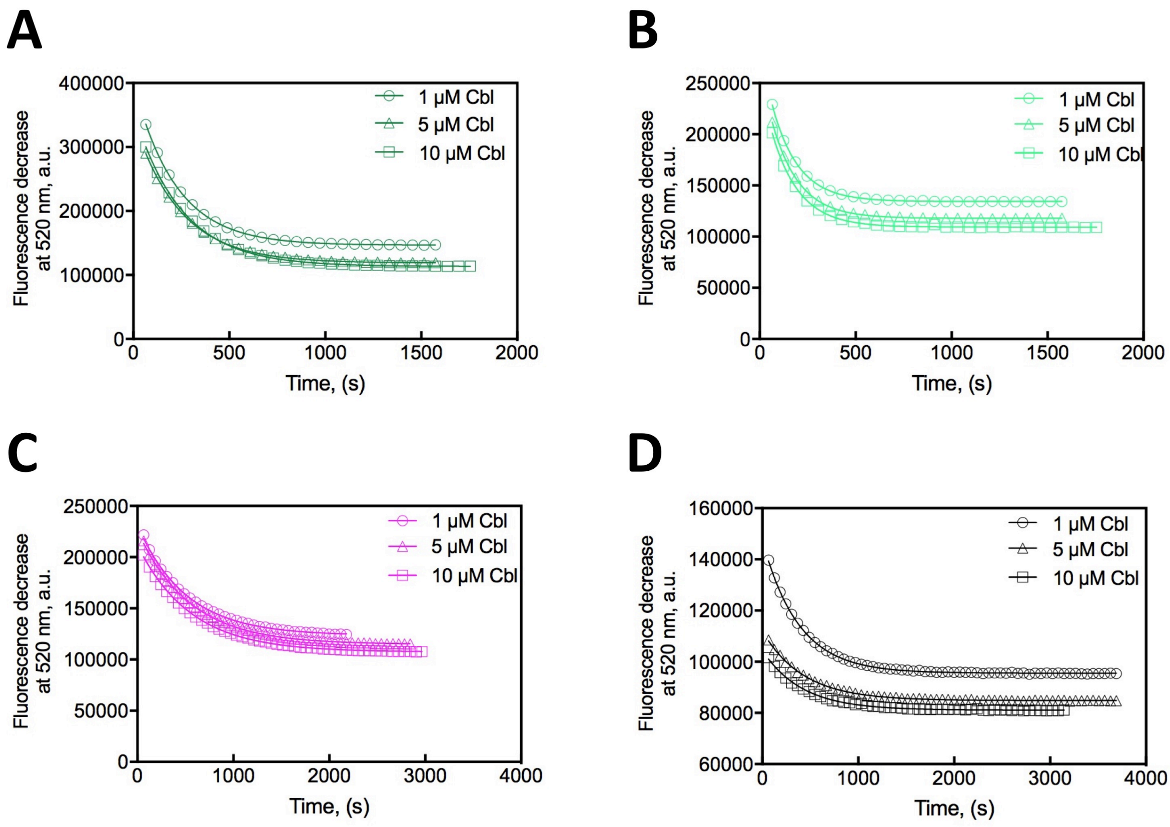

Supplementary Figure 3: Determination of the rates (k_{off}) of spontaneous dissociation of high-affinity nanobodies from BtuF$_{fluo}$ at pH 7.5 and 23 °C. Shown is the raw fluorescence data of the results shown in Figure 3 and Table 1. BtuF$_{fluo}$ (10 nM) was incubated with 10 nM nanobody prior to the addition of Cbl. The resulting solutions of free BtuF$_{fluo}$ and BtuF$_{fluo}$-nanobody complexes were then mixed with different concentrations of excess Cbl (1, 5, or 10 μM) and the kinetics of the decrease in BtuF$_{fluo}$ fluorescence at 520 nm were recorded. The kinetics are characterized by a very rapid fluorescence decrease caused by Cbl binding to free BtuF$_{fluo}$ (finished within the dead time of manual mixing) and a slow phase during which Cbl binds to BtuF$_{fluo}$ molecules that had dissociated from the respective nanobody. All kinetics proved to be independent of Cbl concentration, demonstrating that the slow fluorescence decrease directly monitored nanobody dissociation from BtuF$_{fluo}$. Note that only the kinetics of the slow phases of fluorescence decrease (corresponding to nanobody dissociation from BtuF$_{fluo}$) were evaluated. Shown are the kinetics of dissociation from BtuF$_{fluo}$ for Nb9 (**A**), Nb10 (**B**), Nb7 (**C**) and Nb17 (**D**). The obtained off-rates (mean value of the three rate constants obtained for each nanobody-BtuF complex) are listed in Table 1.

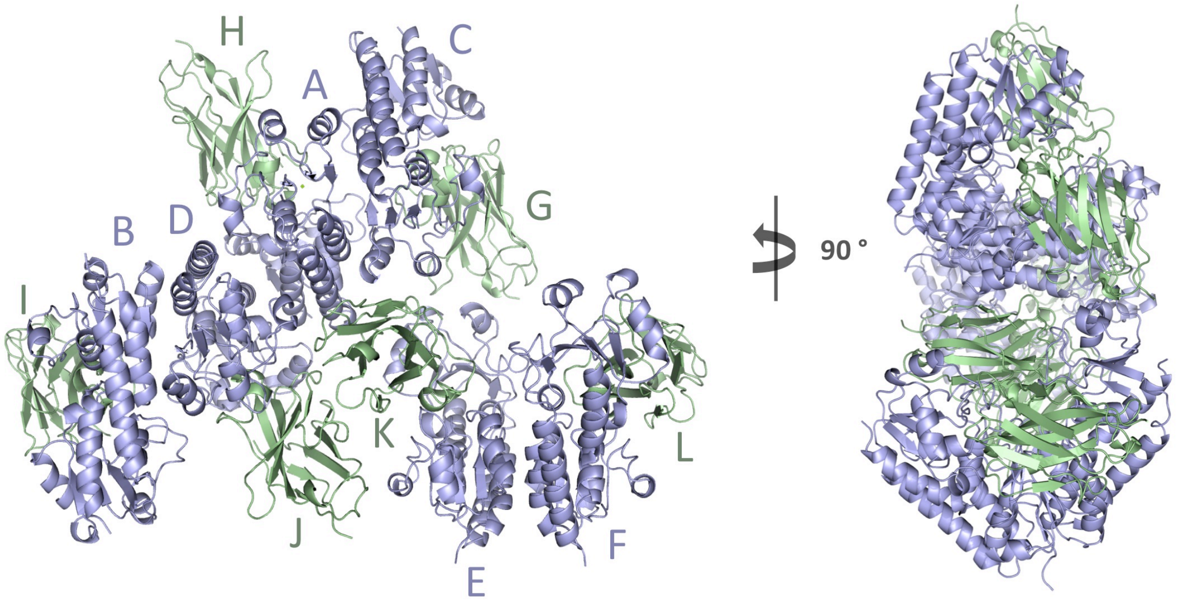

Supplementary Figure 4: Composition of the asymmetric unit. Cartoon representation shows BtuF in light blue (chains A-F) and Nb9 in green (chains G-L). Six Nb9-BtuF complexes were present in the asymmetric unit and ordered in a helix-like arrangement when viewed from the side. The following BtuF and Nb9 chains formed the biological assembly: A + H, B + I, C + G, D + J, E + K, F + L.

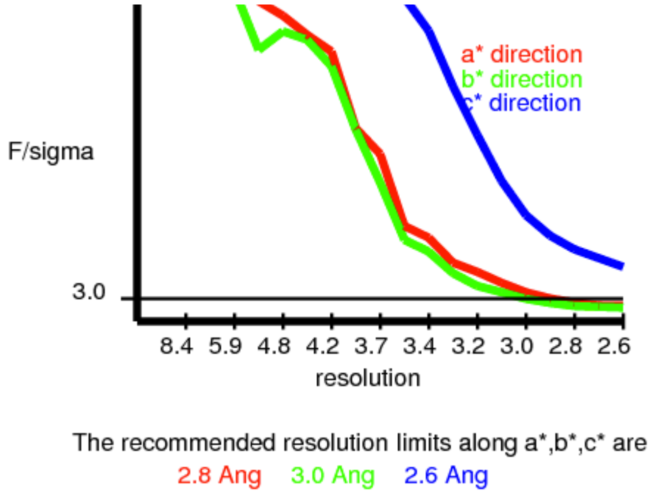

Supplementary Figure 5: Output of the diffraction anisotropy server (UCLA-DOE LAB)[38]. Shown are the recommended resolution limits at which F/sigma drops below an arbitrary cutoff of 3.0.
